# Documentary research on social innovation in health in Latin America

**DOI:** 10.1186/s40249-020-00659-6

**Published:** 2020-04-22

**Authors:** Diana María Castro-Arroyave, Luisa Fernanda Duque-Paz

**Affiliations:** 1 Centro Internacional de Entrenamiento e Investigaciones Médicas (CIDEIM), Cali, Colombia; 2 Icesi University, Cali, Colombia

**Keywords:** Community-based health, Documental research, Public health, Social innovation in health, Social change

## Abstract

**Background:**

Identifying social innovation in health initiatives, promoting quality of life through them, and transforming current health conditions demand the knowledge, comprehension and appropriation of the theoretical and methodological developments of this concept. Academic developments in social innovation have mainly occurred in and been documented for English-speaking countries, although relevant experiences have been implemented in Latin America. In this article, we describe and analyze how social innovation in health is being approached and understood in this region.

**Main text:**

To identify the theoretical and methodological developments of social innovation in health between 2013 and 2018, a scoping review with a mixed approach was carried out. Eighty texts in English, Spanish and Portuguese were selected for a process of reflexive analysis of intra and intertextual reading. The approaches identified in the studied initiatives were complementary. The most applied approaches were innovation in health, technological innovation in health and social innovation, each with twelve publications, and social innovation in health and ecohealth with ten and seven publications respectively. The approaches showed a general interest in reaching the goals of the Sustainable Development Goals (SDGs), the Alma Ata Declaration and the Ottawa Letter.

**Conclusions:**

The social innovation in health approach in Latin America adopts educational strategies, identifies risk factors, optimizes resources, promotes interculturality, participation, community empowerment, and enhances intersectorality and interdisciplinarity. As an approach, process, program or solution, social innovation in health is a conceptual category under construction. This research provides a baseline for other systematic reviews on the subject.

## Background

Most Latin American countries are experiencing social and health problems linked to the past and present conditions of inequality, compounded by the absence of state involvement in the search for solutions to the different problems that afflict populations. Poverty in the region is reflected by the scarcity of goods for subsistence, such as food, shelter, healthcare and drinking water, and the absence of the means to obtain them. Social vulnerability is expressed in extensive squatter settlements that lack basic infrastructure [1], in civil violence and political instability, and in the persistence of social and structural conditions that exclude people from the health system [2]. In addition, interpersonal violence in pervasive, particularly gender-based violence and violence towards children and adolescents, and this is generally linked to “machismo” as an expression of the domination of men and the abuse of masculine power [3]. In summary, structural violence is pervasive [4].

The Pan-American Health Organization (PAHO) [2] has indicated that life expectancy will reach 74.7 years for men and 80.7 years for women in Latin America and the Caribbean by 2030, adding an increasing demand on health systems. Between 2010 and 2015, the rate of teenage pregnancy was the second highest in the world, and this trend has continued. In 2015, health investment was less than 4% of gross domestic product (GDP), public national health expenditure was 3.6% of the GDP, and private health expenditure was 3.4% of the GDP regionwide [2].

Throughout Latin America and the Caribbean, vector-borne diseases such as dengue, malaria, Chagas disease and leishmaniasis are still common, and chronic communicable diseases such as leprosy, human immunodeficiency virus (HIV) and leptospirosis are all also continued health challenges. Many curable conditions are also prevalent: 64 million new cases of sexually transmitted infections (STIs) per year, for example, have been recorded among men and women between the ages of 15 and 49 in this region [5].

These health indices present a challenge to attaining the Sustainable Development Goals (SDGs). The maintenance of good health and prevention of disease needs to be addressed from an integrative perspective, that is, governments and societies should not aim only to reduce disease transmission and severity, but also to identify and tackle potential causes. Given this emphasis on the social determinants of health, states and international organizations are aligning to promote and assume innovative strategies to achieve health and development goals.

Social innovation in health is one approach to this end. The term “social innovation” was coined by Lester Frank Wald in 1908 [6], but it has gained popularity particularly the last 20 years [7, 8] due to its multidisciplinary approach, the versatility in its application, and because it has aroused interest among government decision-makers, non-governmental organizations, researchers, and public and private institutions around the world. The aim of this study is to describe and analyze how social innovation in health is being approached and understood in this region, by identifying its theoretical and methodological developments between 2013 and 2018.

## Methods

### Introduction to the concepts of social innovation and social innovation in health

Social innovation can be conceived as a process, solution, methodology, product, or as a strategy for social change. Its replicable, sustainable and scalable nature requires community involvement in all the stages of the initiative, thus moving from intervention models where solutions of particular needs were in the hands of professionals, to a participatory practice that generates equitable power relations. Authors such as Mason [9] and Mitchell [10] have become important references to understand social innovation and health.

Social innovation in health does neither have a single definition nor a clear role yet due to the lack of scientific and conceptual evidence [11]. However, in Latin America, the Social Innovation in Health Initiative’s (SIHI) definition counts as a starting point. Based on definitions of authors such as Frances Westley and Nino Antadze [12], Geoff Mulgan [13], James A. Phills Jr., Kriss Deiglmeier and Dale T. Miller [8], and Eduardo Pol and Simon Philip Ville [14], social innovation is defined as a novel solution developed in response to a priority health need within a geographical context and implemented by different cross-sectoral organizations. This solution enables more inclusive, affordable and effective health-care delivery. Unlike others, SIHI emphasizes on community-based participation and cross-sectoral interventions.

The SDGs are the global framework for initiatives towards attaining an equitable and sustainable world and social innovation in health is identified as a strategy to achieve such goals while empowering and improving the conditions of neglected communities.

Social innovation in health draws on lessons learned from previous experience to promote better health outcomes. This scoping review’s purpose is to identify the Latin-American theoretical and methodological approaches on social innovation in health between 2013 and 2018 through the following research question: How is social innovation in health being approached and understood in the region?

### Nature of the study

This scoping review is based on an exploratory approach to describe the methodological and conceptual production on social innovation in health in Latin America. To achieve the objective the research performed a search with manual and electronic strategies. The step protocol by Degroote, Bermudez-Tamayo and Ridde (2018) [15] served as guide to our study.

For the analysis process, contributions from quantitative and qualitative approaches were considered [16] in order to reflect the heterogeneity of the documentation and the weave of relationships and thematic connections in the sample, to establish a hierarchical order, identify gaps and articulation needs, and make them visible and accessible so that they can be used by academics and innovators interested in the subject. This involved an iterative process of reflective analysis of intra- and inter-textual reading, as well as reading the descriptive content of various social innovations to detail the approaches and identify contrasts and trends. In our reading of the documentation, we attended to the specificity of particular projects, and to the conceptual and methodological developments and approaches that were described. Descriptive analysis was used to generally characterize the documentary material.

The ideas of the authors and the applications of social innovation in health were analyzed in three methodological moments: a) search for documentary material with defined search criteria, b) classification and selection of the material and, c) quantitative and qualitative analysis of the information.

### Inclusion and exclusion criteria and methodological moments

The initial inclusion criteria for the search were for material published: a) in journals, books and higher degree theses in universities in Latin America; b) in English, Portuguese and Spanish; c) of experiences or theoretical constructs developed in any Latin American country; d) between 2013 and 2018. The corresponding exclusion criteria were: a) documentary material on social innovation with an emphasis on entrepreneurship, b) production from non-Latin American countries, c) documents produced before 2013 and after 2018 and d) material written in languages other than English, Portuguese and Spanish.

This process comprised a series of methodological moments that facilitated systematic work from the search for material to the analysis and communication of results, as presented in the summary below.

### Search for documentary material

The initial search started with the main conceptual category in the three languages of Spanish, English and Portuguese -- “innovación social en salud”, “social innovation in health” and “inovação social em saúde” -- with corresponding filters for year and target countries in article titles, abstracts and keywords. Since the results were few, the descriptors and related concepts were taken from the main conceptual categories – “social innovation”, “health” and “innovation” – which allowed us to identify related concepts to continue the search and so facilitated a more efficient and effective search.

Because the concept of “social innovation” is ambiguous and relatively new in its application, it has not been indexed in different search data bases, even in specialized thesauri such as produced by the United Nations Educational, Scientific and Cultural Organization (UNESCO), the United Nations Bibliographic Information System (UNBIS), and the macrothesaurus of the Organization for Economic Cooperation and Development (OECD).

As a result, the information gathering strategy and text search stage started with setting parameters to select search concepts, with key search terms chosen from preliminary readings in relation to social innovation in health. Our primary task was to identify potential documentation and corroborate its relevance. Accordingly, we proceeded with the search in online databases and institutional repositories selected under the established inclusion criteria. The identification of key concepts and descriptors in the thesauri allowed a search delimitation of 12 descriptors and more than 20 related concepts (Additional file 1).

### Material selection

The search result list was reviewed for the selection of documentary material and registered in Excel matrices and EndNote Web (Clarivate Analytics, Philadelphia, United States) to maintain an organized and systematized record of the documentary material. In this phase, there were about 190 texts. A second review was then conducted by registering the definite documentary material in numerical order in an Excel matrix, with variables identified to allow us to continue with the qualitative and quantitative characterization of the material (Additional file 2).

### Data analysis

Analysis began by recognizing quantitatively and qualitatively what the texts were concerned with, first from the title, abstract and key words, and then by proceeding with the descriptive characterization of the materials, taking into account a series of variables including the sector that produced the publication (academic, governmental, non-governmental organizations, etc.), publication type, authors, place of publication, year of publication, country of publication, country of production or application of experience, language, and most common keywords.

Descriptive analysis of the selected texts led to a definitive discarding of some material, enabling analysis of content from the reading of each text independently (intratextual comprehension), and then a transversal reading to identify converges and divergences between texts of different authors (intertextual comprehension). Excel matrices were used to register the categories found. This allowed us to track the conceptual use of social innovation in health by different authors and innovators.

## Results and discussion

### Leading publishing countries

From the 80 texts that eventually constituted the material relevant for analysis, a general panorama was obtained to characterize developments in the region during the five-year period. This indicated which countries of the region were producing documentary material of their initiatives most actively, which sectors showed most interest in social innovations, which authors were leading and seemed most knowledgeable on the topic, and which institutions played an active role in the production of knowledge.

Social innovation in health initiatives in Latin America were reported and published in scholarly journals from 14 countries, of which 50% are implemented in Latin America. Colombia leads with 25 publications (31.25%) followed by Brazil with 14 (17.5%), Mexico with six (7.5%), Peru with three, Argentina, Honduras and Guatemala with two and Chile, Costa Rica, Venezuela, Uruguay and Paraguay with one publication each. This suggests that in the last 5 years, 12 of 20 Latin American countries (60%) were interested in research in social innovation in health or in innovative ways to improve the services or products for health care. It is possible, however, that there were other initiatives that were not reported in any form, including other countries.

We focus first on the two countries that dominate publications on the subject. It is important to clarify that Colombia has 11 more publications more than Brazil that correspond to undergraduate and postgraduate thesis. Without these 11 publications Colombia and Brazil keep being the publication leaders on the subject, each country with 14 publications. This reflects our greater access to repositories of Colombian universities.

Of the 25 Colombian implementations, 11 were higher degree theses, one a public policy document in relation to science, technology and innovation, 11 were articles published in journals and book chapters, and two reports. If the production is analyzed by number of publications in journals and books or book chapters, Colombia and Brazil share the first place of production and Mexico second. This suggests the particular interest of these three countries in social transformation in health and in building knowledge around new strategies to improve underlying conditions that have an impact on health.

### Approaching the Latin-American concept of “social innovation in health”

From the results of the intra- and inter-textual analysis, it was possible to identify what is understood by social innovation in health in the region, not from the point of view of conceptual development but from the recognition of elements or criteria that characterize it. Lack of conceptual specificity but also interest in the subject suggests that the concept is under construction and instrumental conditions serve as a means, agent or tool crucial to achieve equity and access to health programs and services.

The characteristic criteria of social innovation in health, in part also perceived as a criteria for an intervention to be successful, was identified by authors as that which contributes to empowerment, promotes social value and is owned by the community [17]; contributes to the advancement of the SDGs; contributes to social development; and is adaptable and favors results with less investment than conventional alternatives. Characteristics of these initiatives also include community mobilization and articulation across sectors [18, 19]. Social innovation in health is replicable [18, 20], promotes participation, includes community organizing processes, and has the potential to contribute to the design of a public policy [11]. However, only Rojas, in his work on a state social enterprise attached to a public network in the capital district of Bogotá, made direct reference to “social innovation for health” in the title of the document and in its thematic development [11].

The authors of these articles argued that social innovations in health need to be implemented through intercultural, integrative, environmental and communitarian approaches [17–19, 21]. These include reliance on processes of health education [17, 22], the promotion of research and evidence-based interventions [20, 23], and focus on risk factors [17]. The authors recognized the importance of considering the participation and roles of the community according to gender and age, and tended to adopt a focus on health promotion rather than prevention and treatment of disease. Initiatives must be recursive and resort to existing social, technological, political and community platforms. The case of Guaral/app is illustrative of this approach: the technology enables a presumptive and rapid diagnosis of cutaneous leishmaniasis in distant locations of rural Colombia by non-professional health workers, so benefiting the neglected community of Tumaco [23, 24].

The results, objectives or purposes expected from an implementation of a social innovation in health project refer to: action on the basis of evidence to achieve greater opportunities for scalability and funding proposals that tend to be more effective [23]; contributions to achieve the Millennium Development Goals (MDGs) [18]; seeking sustainability and continuity of programs and processes [22]; achieving applications that reflect evidence about what works and what does not to respond to specific needs or health problems [21]; substantially improving programs to control and manage disease outbreaks [11]; and promoting social value and social and community empowerment (Table 1).

**Table 1:** Main findings on social innovation in health approach characteristics

• Holistic vision	
• Seeks to save costs	
• Adopts technologies or products oriented to solve problems in health	
• Proposes models and strategies, the creation of new capabilities or the rediscovery of forgotten ones	
• Promotes change and sustainability of actions through education and capacity building	
• Promotes intersectorality and multidisciplinarity	
• Recognizes the importance of community participation, empowerment and generates equitable power relations	
• The experiences denote the need to act in situations of social inequity in health	
• No conceptual development was found specifically on the social innovation in health approach	
• There is no clear distinction between technological innovation in health, innovation in health and social innovation in health	

### Identified approaches in the study

Our intra- and inter-textual reading showed the way in which a variety of approaches were made to achieve the goals and address health needs in the region. The initiatives described in the selected material included, in order of higher to lower recurrence: Innovation in Health (12), Technological Innovation in Health (12), Social Innovation (12), Social Innovation in Health (10), Ecohealth (7), Health Equity (1), Social Innovation in Public Health (1), Eco-Bio-Social (1), Frugal Innovation in Health (1), Health Education (1), School Scientific Inquiry (1), and Ecosystem Management (1).

In one particular but relevant way, one author framed the strategy under the approach of social innovation in health in harmony with the SDGs, which articulated with a policy of comprehensive health care and adopted differential approach tools to improve access to health [25]. But greater alignment with social innovation was perceived because the strategy involved the use of a series of practices and processes that sought to form a maternal care network that reduced congestion in obstetric services.

The following describes four of the most common approaches in the analyzed texts:

1. *Innovation in health* is one of the most developed approaches. The authors associated this with features such as: the introduction and intentional application of new ideas, processes, products or procedures within an organization to benefit society in general; initiatives that offer better health care at lower prices [26]; the generation of new medical services [27], new forms of work, new technologies, and new surgical procedures [27, 28]; other health care strategies such as the Unified Health System in Brazil [29]; the implementation of measures to improve access to quality medical services; alternatives to prevent, detect and treat chronic diseases; promotion of the inclusion of people with disabilities; and the centralization of health services [30]. In general, this approach alluded to and often specifically focused on organizational processes to improve health care services and health outcomes.
2. *Technological innovation in health* deals with solutions that aim to improve the efficiency of health services and to create optimal processes which involve the use of technologies to facilitate the work of the health provider [31]. The focus included e-Health, telemedicine [32] and telehealth [33]. The design and use of technological applications are one of the main modalities of this type of innovation.
3. *Social innovation,* although not a category in itself, refers to an initiative in health developed by the authors, mainly in postgraduate theses. None of the published literature or reports that we consulted included social change, social relations or social structures as a focus for innovation.

Two initiatives published with an emphasis on health, “The Park of Life” and “Take a Hand,” were classified as examples of social innovation, and both were included in documentation and analysis by the Latin American Social Innovation Network (LASIN) in 2018. They addressed questions of social inclusion from a comprehensive health perspective, which Puerta describes as “the health of a person in relation to their ability to interact with others and the possibility to thrive in social environments” [34]. In this approach, community participation was prioritized [35], the practices that were implemented were sustainable, and they promoted intersectoral and interinstitutional articulation [34]. These initiatives also promoted empowerment, included participatory methodologies [36], and introduced the territory and population distribution as a variable to help to identify health needs [36]. Another criterion or important characteristic in this approach was the tendency to use novel approaches such as knowledge management to promote social development [37].

4) *Ecohealth* is an approach that studies and manages the relationship between the ecosystem and human systems to promote health. Its main and differentiating attribute is social participation [38], which is aligned with those articles that address Participation as Right and Duty in the Declaration of Alma Ata [38]. Ecohealth also includes education [39, 40], transdisciplinarity, the systemic approach, social equity [38], mainstreaming [41] and the integration of community knowledge [41]; and gives importance to the ecosystem, and includes biological, social, climatic [42] and gender orientation. This approach shares several of the criteria proposed by social innovation in health, and draws attention to the fact that of the seven texts analyzed, six emphasized interventions for vector control; the only one that did not have this emphasis focused on the theoretical development of ecohealth in relation to public health [38]. The one health approach was assimilated with ecohealth by Waleck et al. [40] because it also states that human health cannot be considered in isolation and that people’s health is affected by the ecosystem they inhabit.

Other approaches were less common in the sample, but the authors made important contributions and converged with the criteria promoted by Social Innovation in Health Initiative itself. The *Frugal Innovation in Health* was articulated with cost effectiveness because its purpose was to maximize the value for consumers by offering new products that meet the needs of people at the base of the pyramid (BoP) [43]. The approach of *Education for Health* coincided with the criterion of adaptation and interculturality because it promoted education in non-formal contexts and intervened in health needs identified by communities from an integrative multidimensional and cross-sectional approach. Overall, the approach adopted a health promotion perspective as essential to solve the complex problems that arise in social contexts of vulnerability [44].

*Sustainable and Sanitary Territories* advocated a healthy city, one based on planning and territorial management as fundamental actions for the promotion of health. This approach drew on intersectorality and argued that a greater degree of inclusion and empowerment would facilitate the implementation of social innovation strategies [45]. The health equity approach emphasized the importance of addressing the social determinants of health beyond exclusively clinical settings, and advocated providing access to information and health services to people with a higher risk of morbidity due to poverty and social inequality.

## Approaches and limitations

This article aimed to identify the theoretical and methodological approaches developed in Latin America on social innovation in health between 2013 and 2018. Even though this study reveals how the found initiatives in health were being approached and understood in the region, the great variety of approaches and their dispersive nature make it easy to lose conceptual focus. Adding to this the novelty aspect of social innovation as a field and the attempt to link it to the health field might have helped to broaden the approach results making it difficult to find consistent social innovation in health traits.

In spite of these limitations, the sample of 80 texts identified and selected for a period of 5 years serve as a baseline for other systematic reviews on the subject (Additional file 2). The absence of conceptual developments on social innovation in health limits its existence as a search category in different thesauri. However, the possibility of thinking and tracking other forms of dissemination of innovative health initiatives in the region is open, since those who develop programs or processes of community health intervention are not always familiar with or interested in publishing in academic media. The ‘grey’ literature, forums and web pages may be important sources to identify social innovation initiatives in health.

Social innovation initiatives in health have been published in journals and books in 14 countries, of which seven are in Latin America and the seven remaining countries are United States, Canada, United Kingdom, Switzerland, Germany, Spain and the Netherlands. The Americas and Europe seem to be the continents with the most interest in research publications, with interventions for social transformation in health being documented primarily in Latin America.

The approaches in the selected texts were not exclusive, but complementary. The most frequent cases were innovation in health, technological innovation in health and social innovation, each with 12 publications, with a growing trend in the application of social innovation as a strategy for social development and improvement of health. Social innovation in health (10) and ecohealth (7) were next most common, reflecting current trends in social and health development characterized by implementing comprehensive, multidimensional strategies, multisectoral and multidisciplinary programs that promote social value and social capital. The strategies and initiatives are designed for complex local contexts that face conditions of social inequality and social vulnerability. The approaches are therefore valuable for their general interest in reaching the goals of the SDGs and answering local health needs and problems by promoting horizontal power relations and greater equity in health.

## Conclusions

Social innovation in health is a concept under construction, its definition is incipient, and its foundation lies on social innovation concepts that are further developed. Mainly characterized by interdisciplinarity, intersectorality, and community and social empowerment. Often associated with decision makers, academia and industry but misleadingly affiliated with an entrepreneurial approach.

The reviewed approaches are apt to take on complex and systemic health challenges because of their versatility to simultaneously address social and health needs through strategies that support health, the environment and economic well-being [46].

The flexibility and lack of development of the concept may make it unreliable to identify programs as such because they could be erroneously identified as a health-related artifact, even if they do not comply with the criteria nor had transformative effects. If so, the concept could lose transcendence [47], so it is important to propose ways to distinguish technological innovation in health, innovation in health and social innovation in health from each other.

The sample revealed a marked predominance of experiences compared to conceptual developments, which may respond to matters of urgency and also to the welfare trend that for decades prevailed in the region. This approach promotes self-management and co-management of communities to tackle community passivity [48].

The academy’s calling is to construct knowledge out of previous initiatives to serve as a foundation for future ones, so their role is to become a referent and promote articulation between sectors and disciplines as important elements for sustainability.

The Latin American social context as low- and middle-income countries demand the generation of socially committed community-based initiatives following the social innovation in health approach as proposed by the Hub in Social Innovation in Health for Latin America and the Caribbean, to generate capacity and guarantee the production of knowledge for the design and implementation of initiatives in the region.

## Supplementary information

**Additional file 1.** Descriptors and related terms used for parameterization

**Additional file 2.** Documentary material obtained during the search.

## Data Availability

The authors declare that all material and data used in the manuscript are referenced and freely available online.

## Abbreviations

BoP: People at the base of the pyramid
CIDEIM: Centro Internacional de Entrenamiento e Investigaciones Médicas
HIV: Human immunodeficiency virus
LASIN: Latin American Social Innovation Network
MDGs: Millennium Development Goals
OECD: Economic cooperation and development
PAHO: Pan-American Health Organization
SIHI LAC Hub: Latin American & the Caribbean Hub
SIHI: Social Innovation in Health Initiative
SDGs: Sustainable Development Goals
STIs: Sexually transmitted infections
UNESCO: United Nations Educational, Scientific and Cultural Organization
UNBIS: The United Nations Bibliographic Information System the Organization for Economic Cooperation and Development
