# Supplementary material for: Documentary research on social innovation in health in Latin America
Source: Infect Dis Poverty. 2020 Apr 22;9:41. doi: 10.1186/s40249-020-00659-6 (PMC7175528; doi:10.1186/s40249-020-00659-6)
Supplement: Supplementary file 1 — Additional file 1. Descriptors and related terms used for parameterization [file 40249_2020_659_MOESM1_ESM.pdf]

Additional file 1. Descriptors and related terms used for parameterization

		Thesauri		
Descriptor	***Related Concepts***	***UNESCO***	***OCDE***	***UNBIS***
SOCIAL INNOVATION	SOCIAL INNOVATION	NO	NO	NO
	Social Innovation in Health	NO	NO	NO
INNOVATIONS	INNOVATIONS	NO	YES	NO
	Innovation	NO	NO	NO
	Social Innovation	NO	NO	NO
	Social Transformation	NO	NO	NO
	Social Change	YES	YES	YES
SOCIAL CHANGE	SOCIAL CHANGE	YES	YES	YES
	Social Transformation	NO	NO	NO
	Social Intervention	NO	NO	NO
	Community intervention	NO	NO	NO
	Social Innovation	NO	NO	NO
	Social Appropriation of Knowledge	NO	NO	NO
	Social Development	NO	YES	YES
DEVELOPMENT	DEVELOPMENT	NO	NO	YES
	Social Development	NO	YES	YES
SOCIAL DEVELOPMENT	SOCIAL DEVELOPMENT	NO	YES	YES
	Programs	NO	NO	NO
	Social Transformation	NO	NO	NO
	Community Health	NO	NO	NO
	Social Appropriation of Knowledge	NO	NO	NO
	Social Change	YES	YES	YES
	Health Education	NO	YES	YES
	Projects	NO	NO	NO
TECHNOLOGY TRANSFER	TECHNOLOGY TRANSFER	YES	NO	YES
	Innovation	NO	NO	NO
	Health Technology	NO	NO	NO
	New technologies	NO	YES	NO
	Technology Diffusion	YES	NO	NO
SOCIAL SERVICE	SOCIAL SERVICE(S)	YES	YES	YES
	Projects	NO	NO	NO
	Community Health	NO	NO	NO
	Programs	NO	NO	NO
	Social Intervention	NO	NO	NO
	Community intervention	NO	NO	NO
	Social Appropriation of Knowledge	NO	NO	NO
	Health Education	NO	NO	NO
	Social Change	YES	YES	YES
DEVELOPMENT PROJECT(S)	DEVELOPMENT PROJECTS	YES	YES	YES
	DEVELOPMENT PROJECT	YES	NO	NO
	Experiences	NO	NO	NO
	Project	NO	NO	NO
	Social Transformation	NO	NO	NO
	Social Intervention	NO	NO	NO
	Community intervention	NO	NO	NO
DEVELOPMENT PROGRAM(S)	Development Program	NO	NO	NO
	Development Programs	NO	NO	NO
	Program	NO	NO	NO
SOCIAL PROGRAM	Social Program	YES	NO	NO
	Project	NO	NO	NO
SOCIAL POLICY	SOCIAL POLICY	YES	YES	YES
	Community intervention	NO	NO	NO

	Social Intervention	NO	NO	NO
	Social Transformation	NO	NO	NO
	Social Intervention	NO	NO	NO
	Health Education	NO	YES	YES
HEALTH	HEALTH	YES	YES	YES
	Community Health	NO	NO	NO
	Health Services	YES	YES	YES
	Mental Health	YES	YES	YES
-	TELEMEDICINE	NO	NO	NO
	Telehealth	NO	NO	NO
	Mobile Health Teams	NO	NO	YES
