# Supplementary material for: Documentary research on social innovation in health in Latin America
Source: Infect Dis Poverty. 2020 Apr 22;9:41. doi: 10.1186/s40249-020-00659-6 (PMC7175528; doi:10.1186/s40249-020-00659-6)
Supplement: Supplementary file 2 — Additional file 2. Documentary material obtained during the search. [file 40249_2020_659_MOESM2_ESM.pdf]

Additional file 2. Documentary material obtained during the search

	DOCUMENT TITLE	AUTHOR(S)	TYPE OF PUBLICATION	SEARCH STRATEGY	DATABASE
1	Diseño de una estrategia diferencial para la prestación de servicios de salud de urgencias para la población materna en el Hospital Susana López de Valencia E.S.E.	Beatriz Elena Anacona Zemanate	Master's thesis	University Repository	ICESI University
2	Diagnostics barriers and innovations in rural areas: insights from junior medical doctors on the frontlines of rural care in Peru	Cynthia Fiorella Anticona Huaynate, Monica Jehnny Pajuelo Travezaño, Malena Correa, Holger Mayta Malpartida, Richard Oberhelman, Laura L Murphy and Valerie A Paz-Soldan	BMC Health Serv Res	Specialized database	Web of Science
3	3D printing of cardiac structures: a case of frugal Innovati on in the health sector	Arias, Victor; Contreras-Velásquez, Julio; Chacón, José; Vera, Miguel; Huerfano, Yoleydy; Graterol-Rivas, Modesto; et ál.	Rev Latinoam Hipertens	Specialized database	Redalyc
4	Memoria del foro organizado en el marco de la semana de la innovación en salud	Asociación Mexicana de Industrias de Investigación Farmaceútica	Event report	Search Engine	Google Search
5	Tecnologías de la información y la comunicación (TICs) en el sector salud	Laura Yaneth Avella Martínez; Paola Patricia Parra Ruiz	Postgraduate specialization thesis	University Repository	National University of Colombia
6	Supporting better evidence generation and use within social innovation in health in low- and middle-income countries: a qualitative study	Ballard, Madeleine, Tran, Jenny, Hersch, Fred, Lockwood, Amy, Hartigan, Pamela ; Montgomery, Paul	PLoS One	Specialized database	Web of Science
7	Para mamá: una guía de experiencia maternal	Laura Catalina Baquero Bustos	Undergraduate thesis	University Repository	Andes University
8	Improved dengue fever prevention through innovative intervention methods in the city of Salto, Uruguay	Basso, Cesar, Garcia da Rosa, Elsa Romero, Sonnia Gonzalez, Cristina Lairihoy, Rosario Roche, Ingrid Caffera, Ruben M. da Rosa, Ricardo Calfani, Marisel Alfonso-Sierra, Eduardo Petzold, Max Kroeger, Axel Sommerfeld, Johannes	Trans R Soc Trop Med Hyg	Specialized database	Scopus
9	Telessaúde enquanto instrumento de trabalho do enfermeiro na UTI adulto: uma revisão bibliográfica	Bender, Janaína Duarte Carvalho, Lisa Antunes	J Health Sci	Search Engine	Google Scholar

Additional file 2. Documentary material obtained during the search

**10**	Use of m-health technology for preventive interventions to tackle cardiometabolic conditions and other non-communicable diseases in Latin America-challenges and opportunities	Beratarrechea, Andrea; Diez-Canseco, Francisco; Irazola, Vilma; Miranda, Jaime; Ramirez-Zea, Manuel; Rubinstein, Adolfo	Prog Cardiovasc Dis	Specialized database	Web of Science
**11**	CAPÍTULO II. Contexto Latinoamericano de la Innovación Social<br>2.1 La Innovación Social en América Latina y el Caribe	Bernal, María Elisa	Book chapter	Search Engine	Google Search
**12**	Innovación social en salud: un aporte al avance de los Objetivos de Desarrollo del Milenio	Bernal, María Elisa<br>Robertson, Dustin	Project report	Search Engine	Google Search
**13**	Educación para la salud con adolescentes: un enfoque desde la pedagogía social en contextos y situaciones de vulnerabilidad	Borja González, Juana Del Pozo Serrano, Francisco José	Salud UniNorte	Specialized database	Scopus
**14**	Educación en salud a través del desarrollo de habilidades científicas en escolares chilenos	Soledad Burgos, Karla Yohannessen Andrea Álvarez, Alejandro Rebolledo María Teresa Valenzuela	Salud Publ Mex	Specialized database	Redalyc
**15**	DEPORVIDA Innovando desde el deporte ¿Son las Escuelas Populares del Deporte del INDER Medellín una propuesta de innovación Social? Un análisis entre el año 2001 y 2013	Carlos Alberto Calderón Blandón Yuly Catalina González Ospina	Undergraduate thesis	University Repository	Antioquia University
**16**	Innovación social y desarrollo local. Competencias sociales para la movilización socio/cultural y productiva de las comunidades del departamento del Cauca, Colombia	Yury Yohana Castillo	Master's thesis	University Repository	Cauca University
**17**	Recorrido de participación social desde el enfoque de ecosalud para el control de aedes aegypti. municipio Mario Briceño Iragorry	Ángel Castillo, Ricardo Cornieles Milady Guevara, Milena Mazzarri, Julia Rattia; Karen Flores, Pedro Alcala, Heldomira Guerrero, Marco Marruffo	Comunidad y Salud	Specialized database	Redalyc
**18**	Mosquitiamed: shortening sistances through telemedicine (Gracias A Dios, Honduras)	Castro-Arroyave, Diana María	Web page SIHI	Search Engine	Google Search
**19**	Indigenous community health agents professionalization programme social innovation in health: case studies	Chamas, Liliane,<br>van Niekerk, Lindi	Book chapter SIHI	Search Engine	Google Search

Additional file 2. Documentary material obtained during the search

**20**	Centre for the development of scientific research	Chamas, Liliane,<br>van Niekerk, Lindi	SIHI Article	Search Engine	Google Search
**21**	Innovation in healthcare services: notes on the limits of field research	Costa, Lais Silveira	Cad Saude Publica	Specialized database	SciELO
**22**	Overcoming social segregation in health care in Latin America	Cotlear, Daniel<br>Gómez-Dantés, Octavio<br>Knaul, Felicia<br>Atun, Rifat<br>Barreto, Ivana CHC<br>Cetrángolo, Oscar; et ál	The Lancet	Specialized database	Web of Science
**23**	Auto organización comunitaria para la innovación social y el desarrollo local. Caso comuna 13, Ciudad de Medellín	Cruz Amaya, Margarita María	Master's thesis	University Repository	Pontifical Bolivarian University
**24**	Caracterización de la innovación en el sector de la salud. Estudio de caso	Olga Inés Cuadrado Vega	Master's thesis	Search Engine	Google Search
**25**	Desarrollo de la investigación para la salud en Latinoamérica y el Caribe	Cuervo, Luis Gabriel<br>Clara Bermúdez-Tamayo	Gaceta Sanitaria (Editorial)	Specialized database	Scopus
**26**	Factores que inciden en la aplicación de la innovación social para atender la problemática de obesidad y diabetes en México	de la Calleja, Marco Antonio Lara; Islas, Heidy Cerón<br>Flores, Nora Edith Alcalá, de la Cruz Dorantes, Carlos Román	Strategy, Technology & Society	Search Engine	Google Scholar
**27**	Stories from the field: the use of information and communication technologies to address the health needs of underserved populations in Latin America and the Caribbean	Farach, Nasim ; Faba, Gladys; Julian, Soroya ;Mejía, Felipe; Cabieses, Báltica D'Agostino, Marcelo; Cortinois, Andrea A	JMIR Public Health Surveill	Search Engine	Google Scholar
**28**	Inovação em serviços de saúde no Brasil: análise dos casos premiados no concurso de inovação na administração pública federal	Ferreira, Vicente da Rocha Soares Najberg, Estela<br>Ferreira, Cintia Bragheto<br>Barbosa, Nelson Bezerra<br>Borges, Cândido	Rev Adm Publica	Specialized database	SciELO

Additional file 2. Documentary material obtained during the search

**29**	A tool for exploring the dynamics of innovative interventions for public health: the critical event card	Ana Claudia Figueiro, Sydia Rosana de Araujo Oliveira, Zulmira Hartz Yves Couturier, Jocelyne Bernier Maria do Socorro ,Machado Freire Isabella Samico, Maria Guadalupe Medina, Ronice Franco de Sa, Louise Potvin	Int J Public Health	Specialized database	Web of Science
**30**	Innovative community-based ecosystem management for dengue and Chagas disease prevention in low and middle income countries in Latin America and the Caribbean	Finkelman, Jacobo	Trans R Soc Trop Med Hyg	Specialized database	Scopus
**31**	Governance and the promotion of sustainable and healthy territories: the experience of Bocaina, Brazil	Andréia Faraoni Freitas Setti<br>Helena Ribeiro<br>Ulisses Miranda Azeiteiro<br>Edmundo Gallo	J Integr Coast Zone Manag	Specialized database	SciELO
**32**	La educación del personal de salud:<br>clave para la eliminación de la sífilis congénita en Colombia	Juan Pablo Garcés, Luisa Consuelo Rubiano, Yenifer Orobio, Martha Castaño, Elizabeth Benavides, Adriana Cruz	Biomed	Specialized database	Redalyc
**33**	Oportunidades y retos de la telesalud para la gestión integral de la atención primaria en salud	García Cano, Juan Fernando,<br>Ossa Estrada, Diego Alejandro,<br>Casanova Yela, Rene,<br>Porras Cataño,Sandra Milena,<br>García, Dayhana Alzate,<br>Salazar Marulanda, Nora Luz	Book chapter	Search Engine	Google Search
**34**	Take a hand	Guardatti, Iris<br>Reis, Paulo<br>Monteiro, Guilherme	Book chapter	Search Engine	Google Scholar
**35**	A recombinant live attenuated tetravalent vaccine for the prevention of dengue	Guy, Bruno<br>Noriega, Fernando<br>Ochiai, R. Leon<br>L'Azou, Maina<br>Delore, Valentine<br>Skipetrova, Anna<br>Verdier, Francois	Expert Rev Vaccines	Specialized database	Web of Science
**36**	Community-based global health program for maltreated children and adolescents in Brazil: the equilibrium program	Andrea Horvath Marques, Paula Approbato Oliveira, Luciana Burim Scomparini1, Uiara Maria Rêgo e Silva, Angelica Cristine Silva, Victoria Doretto, Mauro Victor de Medeiros Filho and Sandra Scivoletto	Front Psychiatry	Specialized database	Web of Science

Additional file 2. Documentary material obtained during the search

**37**	Salud: Innovaciones que no sabías que eran de América Latina y el Caribe	Indarte, Selene<br>García Plata, Gabriel Antonio<br>Soto, Alessandra	Book	Search Engine	Google Search
**38**	An Ecohealth approach to fight chagas disease	Irurita, Maria Isabel,<br>Pinto, Lina	SIHI Article	Search Engine	Google Search
**39**	Evaluación de uso del servicio de casa de paso de una EPS indígena en Cali desde la perspectiva del usuario de salud rural	Jiménez Cerquera, Catalina	Master's thesis	University Repository	ICESI University
**40**	Modelo para abordar integralmente la mortalidad materna y la morbilidad materna grave	Karolinski, Ariel;<br>Mercer, Raúl<br>Micone, Paula<br>Ocampo, Celina<br>Salgado, Pablo<br>Szulik, Dalia<br>[illegible]	Rev Panam Salud Publica	Specialized database	SciELO
**41**	Successful malaria elimination in the Ecuador-Peru border region: epidemiology and lessons learned	Krisher, Lyndsay K.; Krisher, Jesse; Ambuludi, Mariano; et ál.	Malar J	Specialized database	Web of Science
**42**	De la participación comunitaria<br>a la participación social: un enfoque de Ecosalud	Roberto Briceño-León Olga B. Ávila Fuenmayor	Espac Abierto	Search Engine	Google Scholar
**43**	Sexual and reproductive health for young adults in Colombia: teleconsultation using mobile devices	Lopez, Catalina Ramirez, Daniel Camilo Valenzuela, Jose Ignacio Arguello, Arturo Saenz, Juan Pablo; et ál.	JMIR Mhealth Uhealth	Specialized database	Web of Science
**44**	Transferencia tecnológica en el área de adicciones: El programa de intervención breve para adolescentes, retos y perspectivas	Kalina Isela Martínez Martínez, María Elena Medina-Mora Icaza	JMIR Mhealth Uhealth	Specialized database	Scopus
**45**	Fitoterapia na Amazônia: a experiência do estado do Amapá-Brasil	Antonio Sergio Monteiro Filocreão, Alexandre Gomes Galindo, Terezinha De Jesus Soares Dos Santos	Book chapter	Search Engine	Google Scholar
**46**	Breeding differently: participatory selection and scaling up innovations in Colombia	Mosquera Vasquez, Teresa; Del Castillo, Sara<br>Cuellar Galvez, David<br>Ernesto Rodriguez, Luis	Potato Res	Specialized database	Web of Science
**47**	Technology for social Inclusion. Recent experiences in Latin America	Muñoz, David Chávez<br>Estebanez, María Elina<br>Ferrero, Aurelio<br>Villalba, Clara	Conference paper	Search Engine	Google Scholar

Additional file 2. Documentary material obtained during the search

**48**	Developing mobile health applications for neglected tropical disease research	Navarro, Andrés<br>Rubiano, Luisa<br>Arango, Juan David<br>Rojas, Carlos A<br>Alexander, Neal<br>Saravia, Nancy Gore<br>Aronoff-Spencer, Eliah	PLoS Negl Trop Dis	Specialized database	Web of Science
**49**	Strategic factors for the sustainability of a health intervention at municipal level of Brazil	Araujo Oliveira, Sydia Rosana; Medina, Maria Guadalupe; Figueiro, Ana Claudia; et ál.	Cad Saude Publica	Specialized database	Web of Science
**50**	Guías bilingües: una estrategia para disminuir las barreras culturales en el acceso y la atención en salud de las comunidades wayuu de Maicao, Colombia	Patiño-Londoño, Sandra Yaneth<br>Mignone, Javier<br>Castro-Arroyave, Diana María<br>Valencia, Natalia Gómez<br>Rojas Arbeláez, Carlos Alberto	Salud Colect	Specialized database	Web of Science
**51**	La construcción de territorio solidario a partir de un enfoque psicosocial desde la atención primaria en salud (APS) y la autogestión comunitaria en la comunidad de Nuevo Occidente, Medellín, Colombia, Sudamérica.	Pérez Villa, Pastor Emilio	Book chapter	Search Engine	Google Search
**52**	Ciencia e innovación tecnológica en la salud en Cuba: resultados en problemas seleccionados	Pérez, Nereida Rojo<br>Pérez, Carmen Valenti<br>Trujillo, Nelcy Martínez<br>Suárez, Ileana Morales<br>Torres, Eric Martínez<br>Estévez, Ileana Fleitas<br>Sao, Miriam Portuondo; et ál	Rev Panam Salud Publica	Search Engine	Google Search
**53**	Comprehensive health approach for Chagas disease in Comapa, Jutiapa - Guatemala	Pinto, Lina<br>Irurita Muñoz, María Isabel	SIHI article	Search Engine	Google Search
**54**	Innovations in Primary Health Care: the use of communications technology and information tools to support local management	Pinto, Luiz Felipe<br>Rocha, Cristianne Maria Famer	Cien Saude Colet	Specialized database	Web of Science
**56**	Análisis de la propuesta de modelo social de la granja Tarapacá para la inclusión social de las personas con discapacidad intelectual	Lizeth Ramón Palacio	Master's thesis	University Repository	ICESI University

Additional file 2. Documentary material obtained during the search

**57**	Ciencia, tecnología e innovación para la salud. Lineamientos de Política 2017- 2020	Alcaldía Mayor de Bogotá	Government Document	Search Engine	Google Search
**58**	Medellín y el sueño de ciudad saludable: construcción técnica y colectiva	Jairo H. Restrepo-Zea, Eliana Martínez-Herrera y Andrea Ruiz-Molina	Rev Sal Publica	Specialized database	PubMed
**59**	The role of gender in Chagas disease prevention and control in Honduras: an analysis of communication and collaboration networks	Rodriguez Triana, Diana RocioMertens, Frederic<br>Valeriano Zuniga, Concepcion<br>Mendoza, Yolanda<br>Nakano, Eduardo Yoshio<br>Carlota Monroy, Maria	Ecohealth	Specialized database	Scopus
**60**	Caracterización de las iniciativas en innovación social para la salud de un empresa social del estado adscrita a la red pública del Distrito	Rojas Cárdenas, A.	Gray literature	Search Engine	Google Search
**61**	Casos de innovación social en salud en Colombia: retos y proyectos	Ruíz Ibañez, Carlos	Rev ing biomed	Specialized database	SciELO
**62**	A obesidade extremamente pobre: contribuições das ciências sociais para as políticas públicas de alimentação e nutrição	Daniela Sanches Frozi Co-autores: Denise Oliveira Caio Capella	Conference paper	Specialized database	Google Scholar
**63**	Revisión sistemática: modelos innovadores disruptivos en la prestación de los servicios de salud entre los años 2004 y 2014	Pereira Santiago, Fernando<br>Camacho Mantilla, Julio César	Master's thesis	University Repository	Pontifical Xavierian University
**64**	Ecosystem research experience with two indigenous communities of Colombia: the ecohealth calendar as a participatory and innovative methodological tool	Andrés Felipe SantoDomingo, Laura Castro-Diaz, Catalina González-Uribe<br>The Wayúu Community of Marbacella and El Horno and The Barí Community of Karikachaboquira	Ecohealth	Specialized database	Scopus
**65**	Telemedicine: challenges to dissemination in Brazil	Maldonado, Jose Manuel Santos de Varge; Marques, Alexandre Barbosa; Cruz, Antonio	Cad Saude Publica	Specialized database	Web of Science

Additional file 2. Documentary material obtained during the search

**66**	A score to predict and stratify risk of tuberculosis in adult contacts of tuberculosis index cases: a prospective derivation and external validation cohort study	Saunders, Matthew J<br>Wingfield, Tom<br>Tovar, Marco A<br>Baldwin, Matthew R<br>Datta, Sumona<br>Zevallos, Karine; et ál.	Lancet Infect Dis	Specialized database	Web of Science
**67**	O cuidado ao usuário de crack: estratégias e práticas de trabalho no território	Aline Basso da Silvaa Leandro Barbosa Pinhoa<br>Agnes Olschowskya Débora Schlotefeldt<br>Siniaka Cristiane Kenes Nunesa	Rev Gaucha Enferm	Specialized database	PubMed
**68**	Innovative community-based vector control interventions for improved dengue and Chagas disease prevention in Latin America: introduction to the special issue	Sommerfeld, Johannes<br>Kroeger, Axel	Trans R Soc Trop Med Hyg	Specialized database	Scopus
**69**	Family health strategy: a technological innovation in health	Soratto, Jacks; Pires, Denise Elvira Pires de, Dornelles, Soraia; Lorenzetti, Jorge	Texto Contexto Enferm	Specialized database	SciELO
**70**	Innovación social para mejorar la calidad de vida del adulto mayor: estudio de caso del programa centro día en Santiago de Cali	Paola Andrea Suarez Molano	Master's thesis	University Repository	ICESI University
**71**	Desarrollo de un sistema georreferenciado para la gestión, movilidad y monitoreo de atención primaria de la salud comunitaria	Tetamanti, Juan Manuel Diez;<br>Rocha, Eduardo<br>Munsberg, Glauco<br>Peixoto Castro, Jessica Helena<br>Neutzling, Aline dos Santos<br>Jaime, Sergio Fernando<br>Schuler, Leonardo Javier	Salud Colect	Specialized database	SciELO
**72**	Web-based intervention to reduce substance abuse and depressive symptoms in Mexico: development and usability test	Tiburcio, M.<br>Lara, M. A.<br>Abrego, A. A.<br>Fernandez, M.<br>Velez, N. M.<br>Sanchez, A.	JMIR Ment Health	Specialized database	Scopus
**73**	Revisiting the concept of innovative developing countries (IDCs) for its relevance to health innovation and neglected tropical diseases and for the prevention and control of epidemics	Vasconcellos, A. G.<br>Fonseca, Bdfe<br>Morel, C. M.	PLoS Negl Trop Dis	Specialized database	Web of Science

Additional file 2. Documentary material obtained during the search

**74**	Mejoramiento barrial y urbano en costa rica: reconstrucción social de la salud comunitaria desde la perspectiva de salud ambiental	Christiam Álvarez Vega	Poblac Salud Mesoam	Specialized database	SciELO
**75**	Aplicación de la eSalud en el contexto cubano	Vidal, M	Rev Panam Salud Publica	Search Engine	Google Scholar
**76**	Panorama actual de la innovación social en Colombia	Laura Villa<br>Jenny Melo	Project report	Search Engine	Google Search
**77**	An innovative ecohealth intervention for Chagas disease vector control in Yucatan, Mexico	Etienne Waleckxa, Javier Camara-Mejiaa, Maria Jesus Ramirez-Sierraa, Vladimir Cruz-Chana, Miguel Rosado-Valladoa, Santos Vazquez-Narvaezb, Rosario Najera-Vazquezb, Sebastien Gourbie`rec and Eric Dumonteila.	Trans R Soc Trop Med Hyg	Specialized database	Web of Science
**78**	Non-randomized controlled trial of the long- term efficacy of an Ecohealth intervention against Chagas disease in Yucatan, Mexico	Etienne Waleckx, Silvia Pérez-Carrillo, Samuel Cháez-Lazo, Rafael Pasos-Alquicira, María Cámara-Heredia, Jesu Acuña-Lizama1, Fernando CollíBalá m; et ál.	PLoS Negl Trop Dis	Specialized database	Scopus
**79**	Annual Report	Tropical Disease Research	Project report	Search Engine	Google Search
**80**	Crowdsourcing in health research SIHI	World Health Organization 2018	Project report	Search Engine	Google Search
